# Oocyte Arrested at Metaphase II Stage were Derived from Human Pluripotent Stem Cells *in vitro*

**DOI:** 10.1007/s12015-023-10511-7

**Published:** 2023-02-03

**Authors:** Xiaoli Yu, Ning Wang, Xiang Wang, Hehe Ren, Yanping Zhang, Yingxin Zhang, Yikai Qiu, Hongyan Wang, Guoping Wang, Xiuying Pei, Ping Chen, Yahui Ren, Chunfang Ha, Li Wang, Huayan Wang

**Affiliations:** 1 Key Laboratory of Fertility Preservation and Maintenance of Ministry of Education, School of Basic Medical Sciences, Ningxia Medical University, 750004 Yinchuan, Ningxia China; 2 Department of Animal Biotechnology, College of Veterinary Medicine, Northwest A&F University, 712100 Yangling, Shaanxi China; 3 Department of Gynecology, General Hospital of Ningxia Medical University, Ningxia Human Sperm Bank, 750004 Yinchuan, Ningxia China; 4 Yinchuan Maternal and Child Health Care Hospital, 75004 Yinchuan, China; 5 College of Life Science and Engineering, Henan University of Urban Construction, 467000 Pingdingshan, China

**Keywords:** Human pluripotent stem cells, Differentiation, Follicle fluid, Follicle-like structures, Oocyte-like cells

## Abstract

Initiation of meiosis is the most difficult aspect of inducing competent oocytes differentiation from human stem cells *in vitro*. Human induced pluripotent stem cells (hiPSCs) and embryonic stem cells (hESCs) were cultured with follicle fluid, cytokines and small molecule to induced oocyte-like cells (OLCs) formation through a three-step induction procedure. Expression of surface markers and differentiation potential of germ cells were analyzed *in vitro* by flow cytometry, gene expression, immunocytochemistry, western blotting and RNA Sequencing. To induce the differentiation of hiPSCs into OLCs, cells were firstly cultured with a primordial germ cell medium for 10 days. The cells exhibited similar morphological features to primordial germ cells (PGCs), high expressing of germ cell markers and primordial follicle development associated genes. The induced PGCs were then cultured with the primordial follicle-like cell medium for 5 days to form the induced follicle-like structures (iFLs), which retained both primordial oocytes-like cells and granulosa-like cells. In the third step, the detached iFLs were harvested and transferred to the OLC-medium for additional 10 days. The cultured cells developed cumulus-oocyte-complexes (COCs) structures and OLCs with different sizes (50–150 μm diameter) and a zona pellucida. The *in vitro* matured OLCs had polar bodies and were arrested at metaphase II (MII) stage. Some OLCs were self-activated and spontaneously developed into multiple-cell structures similar to preimplantation embryos, indicating that OLCs were parthenogenetically activated though *in vitro* fertilization potential of OLCs are yet to be proved. *in vitro* maturation of OLCs derived from hiPSCs provides a new means to study human germ cell formation and oogenesis.

**Graphical Abstract:**

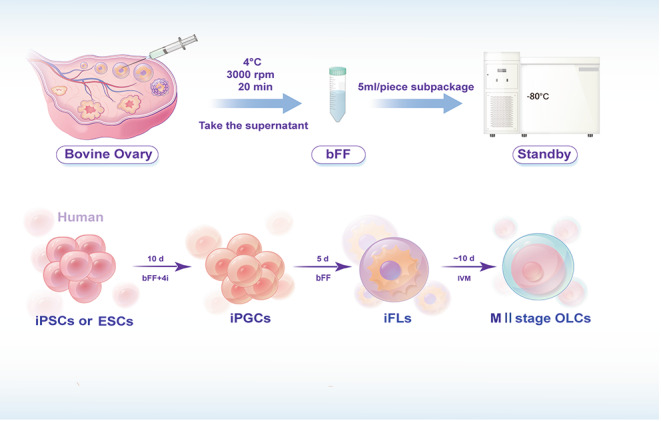

**Supplementary Information:**

The online version contains supplementary material available at 10.1007/s12015-023-10511-7.

## Introduction

Understanding the full process of gametogenesis *in vitro* requires the generation and culture of germ cells in the absence of the morphogenetic events of gastrulation. Compelling evidence indicates that human and mouse pluripotent stem cells can generate oocyte-like cells (OLCs) to mimic oogenesis *in vitro* [1–6]. Suspension culture is commonly used to induce pluripotent stem cells differentiation into primordial germ cells (PGCs) [1], which were continuously grown in media containing cytokines such as BMP4, SCF, EGF, and LIF, and aggregated with female gonadal somatic cells as reconstituted ovaries to induced oogenesis and folliculogenesis *in vitro* or *in vivo* [4, 7, 8]. Meanwhile, OLCs have also been produced from somatic stem cells and induced under monolayer culture conditions in a conditional medium enriched with porcine follicular fluid and ovarian steroid hormones [9–11]. A mature oocyte was successfully reconstituted from mouse ESCs, which developed into PGCs coupled with somatic cells from embryonic day 12.5 (E12.5) female gonad [12]. Oogonia-like cells produced from human pluripotent stem cells were recently reconstituted with mouse embryonic ovarian somatic cells to form xenogeneic ovary, which harbored germ cells with epigenetic reprogramming and entered meiotic prophase [4]. However, variations in germ cell development between mammalian species were evident [13, 14]. A recent study found that overexpression of DAZL and BOULE with recombinant GDF9 and BMP15 (also known as GDF9B) induced human ESCs to enter meiosis and form follicle-like structures (FLs) *in vitro*, in which the mixed cells of OLCs and granulosa-like cells were self-organized and resembled the primary follicles [3].

Our earlier study showed that human amniotic fluid stem cells (hAFSCs) could produce primordial follicles a two-step procedure, but they could not be stimulated to develop into antral follicles to form metaphase II (MII) oocytes [9]. Here, we demonstrated that MII OLCs were produced from human iPSCs and ESCs with considerably higher self-renewal and differentiation potential than hAFSCs in adherent monolayer culture in roughly 30 days using a three-step approach without constructing artificial ovary. MII OLCs derived from hPSCs offer a novel approach to investigating human folliculogenesis and oogenesis.

## Materials and Methods

### Cell Culture

Human induced pluripotent stem cells (hiPSCs, female XX, TAC153) and human embryonic stem cells (hESCs, female XX, H9) were purchased from SiDanSai Biotechnology Co. and ATCC, respectively. The hPSCs were cultivated in mTeSR™1 (Stem Cell Technology) on Matrigel (Becton Dickinson) until 80% confluence. A three-step induction procedure was conducted to generate OLCs.

**Step 1.** Formation of induced PGC cells (iPGCs, day 0 to day 10). The hPSCs were cultured in a PGC-m medium for 10 days to generate iPGCs. The PGC-m medium was constituted as follows: alpha minimum essential medium (alpha-MEM; Gibco) supplemented with 5% knockout serum replacement (KSR; Gibco), 5% bovine follicular fluid (bFF), 1% L-glutamine (Invitrogen), 1% nonessential amino acids (NEAA; Gibco), 0.1 mM β-mercaptoethanol (β-ME, Sigma), 1% penicillin/streptomycin, 50 ng/mL BMP4 (PeproTech), 200 ng/mL human LIF (Sino Biological), 100 ng/mL SCF (PeproTech), 50 ng/mL EGF (stemRD), and 10 µM ROCK inhibitor (Y-27,632, Selleck). The hPSCs were cultured in PGC-m at 37 °C, 5% CO_2_ in a 3 × 10^5^ /3.5 cm plate pre-coated with Matrigel. The medium was replenished daily. The hPSCs developed a flattened epithelioid morphology with distinct cell-to-cell boundaries on day 2 post-induction. SOX17-positive cell clusters (25 to 50 μm in diameter) emerged progressively throughout differentiation. Cell aggregates and induced primordial germ cells (iPGCs) appeared on the top of the monolayer cells on days 8–10 post-induction.

**Step 2**. Formation of induced follicle-like structures (iFLs, day 11 to day 15). The iPGCs were cultured in a follicle-like cell culture medium (FLC-m) for 5 days to generate iFLs. FLC-m medium is composed of alpha-MEM supplemented with 5% KSR, 5% bovine follicle fluid (bFF), 1% L-glutamine, 1% NEAA, 0.1 mM b-ME, and 1% penicillin/streptomycin. Cell aggregates were then incubated in FLC-m for an additional 5 days after day 11 of induction. Half of the liquid volume medium was changed into the medium, every day. Induced follicle-like structures (iFLs) with diameter from 50 to 200 μm were generally evident under the microscope on experiment day 15.

**Step 3.** Formation of oocyte-like cells (OLCs, day 16 to day 25). iFLs were collected, transferred into OLC-m, and cultured for additional 10 days for OLC *in vitro* maturation. In brief, iFLs were transferred into a 30 µL droplet, coated with mineral oil, and cultured in OLC-m for 10 to 15 days, with half of the medium changed every 2 days. OLCs with diameter ranging from 50 to 150 μm could be observed. OLC-m medium is composed of TCM 199 (Gibco) supplemented with 3 mg/mL bovine serum albumin (BSA, Sigma), 5 U/mL follicle-stimulating hormone (FSH, Sigma), 10 U/mL human chorionic gonadotropin (hCG, Sigma), 10 IU/mL pregnant mare serum gonadotropin (PMSG, Sigma), 0.23 mM pyruvic acid, 10 ng/mL epidermal growth factor (EGF, Sigma), and 1% insulin-transferrin-selenium (ITS, Gibco).

Table S1 lists all media used in this investigation. bFF was prepared as follows: the bovine ovaries from mature cows were collected from a licensed local slaughterhouse, Sanqiao Slaughterhouse in Xi`An, China, and transported to the laboratory in sterile saline at 37 °C within 6 h. The bFF was aspirated from follicles using a 10-mL syringe with an 18-gauge needle. The cumulus-oocyte complexes (COCs) and granular cells were removed from bFF by centrifugation at 3000 g for 20 min at 4 °C. The bFF was filtered through a 0.22-µm sterile filter, aliquoted in a 5 mL/tube, and stored at -80 °C for subsequent use.

### iPGCs Purification

iPGCs were purified from 7-day differentiated cell aggregates derived from hiPSCs by immunomagenetic isolation of DEAD-box helicase 4 (DDX4; also known as vasa) antibody. The iPGC cells were washed 3 times in PBS, placed into PBS containing 0.05% trypsin, and incubated for 5 min at 37 °C, 5% CO_2_. Trypsin was neutralized using a culture medium containing 10% FBS. The dispersed cells were carefully collected and centrifuged at 1000 g for 5 min, the pellet was resuspended and washed twice with PBS. The cells were resuspended and conjugated with DDX4 antibody pre-coated with goat anti-rabbit IgG microbeads. DDX4^+^ iPGCs were separated magnetically, according to manufacturer’s instruction.

### Alkaline Phosphatase Staining

Alkaline phosphatase staining was performed using the Alkaline Phosphatase Assay Kit (P0321S, Beyotime) according to the manufacturer’s instruction.

### iPGCs Culture

The DDX4 antibody purified PGCs at a density of 5,000 cells were seeded in a 6-well plate containing mitotically inactivated STO feeder layers. The medium was constituted as follows: Minimum essential medium alpha with 10% FBS, 1 mM sodium pyruvate (Sigma), 1 mM NEAA, 2 mM L-glutamine (Sigma), 0.1 mM β -mercaptoethanol (Sigma), 10 ng/mL human leukemia inhibitory factor (LIF; Santa Cruz Biotechnology, sc-4377), 10 ng/mL human epidermal growth factor (EGF; Peprotech, AF-100-15), 40 ng/mL human glial cell line-derived neurotrophic factor (GDNF; Peprotech, 450 − 10), 10 ng/mL human basic fibroblast growth factor (bFGF; Peprotech, AF-100-18B), and 6 mg/L penicillin [15]. The medium was changed daily.

### Plasmid Transfection

The pBMP15-EGFP vector carrying the human BMP15 promoter sequence was constructed as previously described [16]. For transient transfection, the differentiated cells were washed once with Dulbecco’s phosphate-buffered saline (DPBS, Gibco) and then transfected with 4 µg pBMP15-EGFP plasmid using Lipofectamine 3000 Reagent (Invitrogen) according to the manufacturer’s introduction. Images were captured with a fluorescence microscope (Nikon).

### Flow Cytometry

The iPSCs and 10-day differentiation iPGCs were treated with Accutase (Invitrogen), rinsed with culture medium to assist in washing out cells, and pipetted gently up and down. Following a single wash with DPBS containing FBS and 0.1% BSA, the cell suspension was filtered through a 70 μm cell strainer (BD Biosciences) to remove cell aggregates and centrifuged. Cells were treated with the anti-c-KIT antibody (#562,094, BD Biosciences) for 15 min on ice. Nonbinding isotype-matched FITC-conjugated mouse IgG was used in control assays. The cells were suspended in FACS buffer (0.1% BSA in DPBS) and examined using a FACSCalibur Flow Cytometer (BD Biosciences, USA).

### Gene Expression Analysis

Total RNAs were extracted by RNeasy Mini Kit (Qiagen) following the manufacturer’s introduction. Following that, 1 µg of RNA was reverse transcribed into cDNA by RevertAid™ kit (Thermo Fisher Scientific) and used to conduct PCR in 50 µL reaction volumes for 35 cycles. The GAPDH expression level served as the internal control. Quantitative RT-PCR (qRT-PCR) was performed using the SYBR Premix Ex TaqTM kit (TaKaRa) on a programmed thermal cycler (ABI), following the manufacturer’s protocol. Target genes expression levels were quantified by the 2^−ΔΔCT^ method using GAPDH as internal control. Human ovary tissue served as the positive control sample. Table S2 lists the primers used in this study.

### Immunocytochemistry and Western Blotting

Protein analysis was performed as previously described [16]. First, immunocytochemistry staining was performed as follows: differentiated cells from days 0, 5, 10, 15, 25 and 35 were fixed in 4% paraformaldehyde in DPBS for 20 min at room temperature. The fixed cells were then washed twice with DPBS containing 0.2% Triton X-100 for 10 min at room temperature. Cells were blocked with blotting buffer (1% BSA, 0.1% Tween 20 in DPBS) for 2 h, and then treated with the primary antibody at 4 °C overnight. Antibodies against OCT4 (1:300, SC-5279, Santa Cruz), SOX2 (1:400, #3579, Cell Signaling Technology), PRDM1 (1:300, SC-47,732, Santa Cruz), SOX17 (1:300, SC-130,295, Santa Cruz), STELLA (1:500, ab74531, Abcam), FRAGILIS (1:300, ab15592, Abcam), NOBOX (1:400, ab41521, Abcam), DDX4 (1:300, ab13840, Abcam), BMP15 (1:500, PA5-34401, Thermo Fisher), ZP3 (1:300, 23,715, Santa Cruz), FOXL2 (1:400, NB100-1277, Novus Biologicals), SCP3 (1:500, ab181746, Abcam), ZP2 (1:300, 32,894, Santa Cruz), c-KIT (1:500, ab5505, Abcam), CX37 (1:300, ab83788, Abcam), and α-Tubulin (1:400, ab18251, Abcam) were used. The same batch of differentiated cells was treated with isotype-matched IgG and used as a control. After three washes, the cells were incubated with a secondary antibody for 1 h at room temperature. The cells were then washed as described above, and cellular nuclei were stained with DAPI dye at a concentration of 1 µg/mL for 5 min, and re-washed as described above, human immature oocytes that were discarded during the IVF program were collected and used as controls and visualized using a fluorescence microscope or confocal microscope. Western blot analysis was performed as previously described [16]. The differentiated cells were collected on days 0, 5, 10, 15, and 25 following a washing step with cold DPBS and treated with 200 µL RIPA buffer (50 mM Tris, 150 mM NaCl, 1% NP-40, 0.5% Sodium deoxycholate, 0.1% SDS, pH 8) with protease inhibitors (1 mM PMSF). The human ovary was employed as a control. Protein concentration was measured in the supernatant, heated at 100 °C for 5 min, and loaded onto 10% SDS-PAGE. The proteins were subsequently transferred to a PVDF membrane (LC2002, Invitrogen) using semi-dry electrophoretic transfer (Bio-Rad) at 15 V for 45 min. The membrane was blocked with 5% non-fat milk for 1 h at room temperature and then incubated with primary antibodies, including anti-OCT4 (1:500, SC-5279, Santa Cruz), anti-BMP15 (1:1000, ab108413, Abcam), anti-SCP3 (1:1000, ab181746, Abcam), anti-ZP3 (1:300, SC-398,359, Santa Cruz), anti-β-Actin (1:1000, TA-09, ZSGB-BIO) and anti-GAPDH (1:2000, KM9002, Sungene Biotech), for 2 h. Following three washes with TBS-T buffer (20 mM Tris/HCl pH 7.6, 137 mM NaCl, 0.1% Tween 20), the membrane was incubated with an HRP-conjugated secondary antibody (1:3000, A0258, Beyotime) at 37 °C for 1 h. The membrane was incubated in the enhanced chemiluminescent substrate (#32,106, Pierce) for 1 min after three washes in TBST (Tris-buffered saline, 0.1% Tween 20) at room temperature, and examined with a Chemiluminescent Imaging System (ZY058176, Tanon-4200).

### Light Microscopy

Follicle-like structures were selected and fixed in 0.1 M phosphate buffer containing 2% glutaraldehyde. Following several washes with 0.1 M phosphate buffer, FLs were post-fixed in 1% osmium tetroxide, rinsed with 0.1 M phosphate buffer, dehydrated in increasing concentrations of ethyl alcohol (30, 50, 70, 80, 90, and 2 × 100%), infiltrated with epoxy resin overnight, and polymerizing overnight at 60 °C. To evaluate the follicular structure, 1 μm thick epoxy sections were stained with 1% (w/v) aqueous methylene blue and examined using an Olympus BX50 microscope.

### RNA Sequencing

hiPSCs were seeded onto a six-well-plate coated with Matrigel, each well of iPGCs was lysed in 1mL TRIzol reagent (Invitrogen, 15596018) after 5 days differentiation in three independent experiments, and hiPSCs were used as control. The RNA sample preparations required 3 µg RNA per sample. Sequencing libraries were generated using NEBNext® UltraTM RNA Library Prep Kit for Illumina® (NEB, USA) following the manufacturer’s guidelines, and index codes were added to assign sequences to each sample. Briefly, mRNA was purified from total RNA using poly-T oligo-attached magnetic beads. Fragmentation was performed using divalent cations under elevated temperature in the NEBNext First Strand Synthesis Reaction Buffer (5X). First-strand cDNA was synthesized using random hexamer primer and M-MuLV Reverse Transcriptase and second-strand cDNA was synthesized using DNA Polymerase I and RNase H. The remaining overhangs were converted into blunt ends via exonuclease/polymerase activities. The 3’ ends of DNA fragments were adenylated, and the NEBNext Adaptor with hairpin loop structure was ligated in readiness for hybridization. The library fragments were purified with the AMPure XP system (Beckman Coulter, Beverly, USA) to select cDNA fragments of preferably 250 ~ 300 bp length. After that, 3 µl USER Enzyme (NEB, USA) was incubated with size-selected, adaptor-ligated cDNA at 37 °C for 15 min, followed by another 5-min incubation at 95 °C before PCR. The PCR was performed using Phusion High-Fidelity DNA polymerase, Universal PCR primers, and Index (X) Primer. Finally, PCR products were purified (AMPure XP system) and the quality of the library was evaluated using the Agilent Bioanalyzer 2100 system. The index-coded samples were clustered using the cBot Cluster Generation System using TruSeq PE Cluster Kit v3-cBot-HS (Illumia) according to the manufacturer’s instructions. The library preparations were sequenced on an Illumina Hiseq platform and 125 bp/150 bp paired-end reads were generated, and the multiplexed library was sequenced on an Illumina Hiseq 2000 platform according to the manufacturer’s recommendations (Illumina) at Novogene Bioinformatics Institute, Beijing, China.

### Statistical Analysis

All data were presented as mean ± SD. Statistical analyses of Student’s *T*-test were performed using GraphPad Prism software, as illustrated in the figure legends. *P* < 0.05 and *P* < 0.01 were regarded as statistically significant and highly significant, respectively. Conclusions were reached after at least three independent experiments.

## Results

### Derivation of iPGCs from Human PSCs

**Fig. 1:**
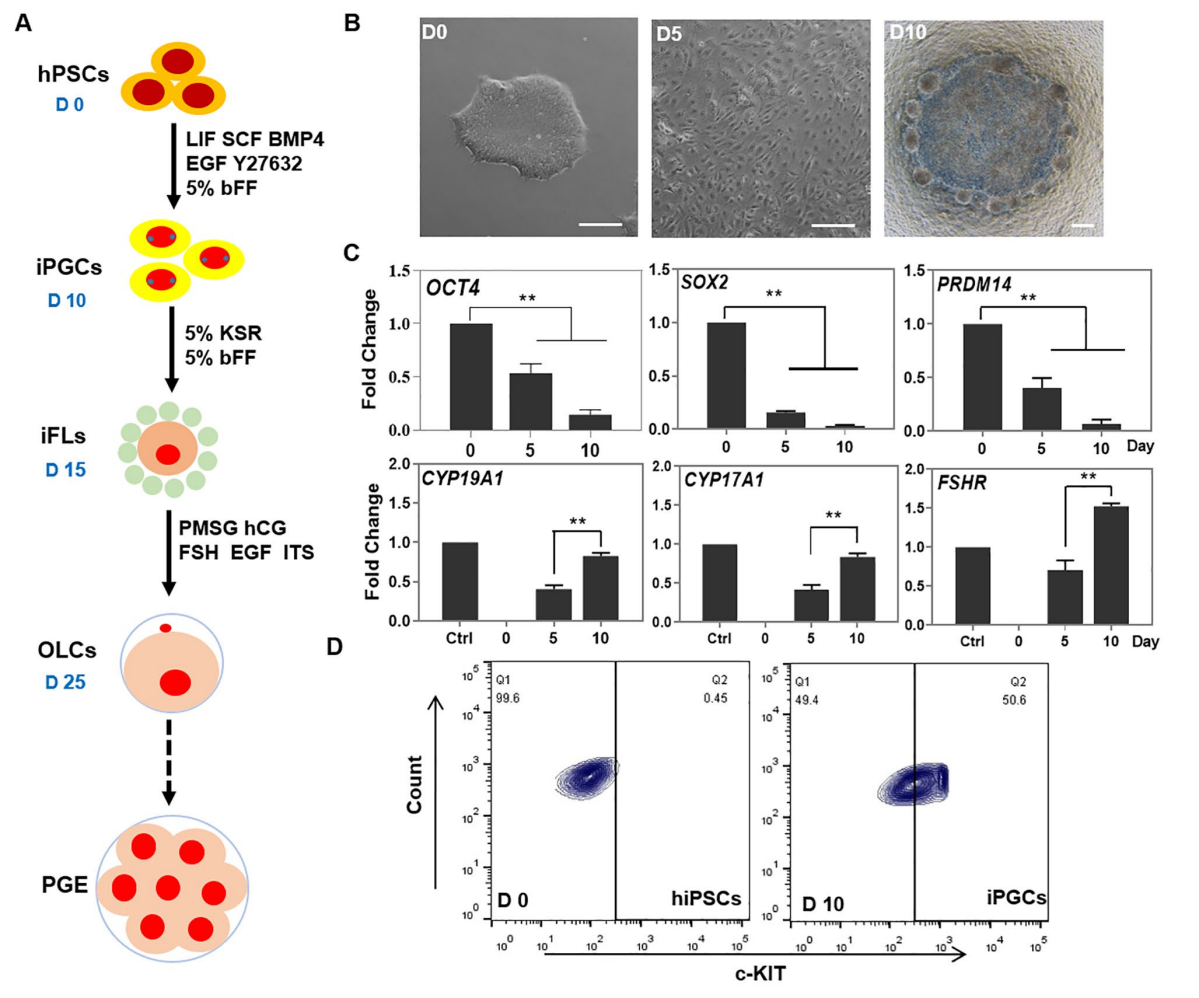
Differentiation of hiPSCs into early stage of iPGCs. (A) Schematic diagram of OLCs derived from hPSCs. (B) Morphology of early stage of iPGCs derived from hiPSCs at day 10 (D10). Scale bar, 200 μm. (C) qRT-PCR analysis of pluripotent genes (*OCT4*, *SOX2*, and *PRDM14*) and hormone-related genes (*CYP19A1*, *CYP17A1*, and *FSHR*) in iPGCs. Ctrl, human ovary tissue was used as positive control. (D) Flow cytometry analysis of c-KIT expression in hiPSCs and early stage of iPGCs at day 10. Data indicate mean ± SD. **P < 0.01, n = 3

hESCs and hiPSCs have primed pluripotency (Fig. S1A-B), as such, we investigated their ability to differentiate into iPGCs using the approach shown in Fig. 1A. After 10 days of culture in PGC medium (PGC-m), the morphology of hESCs and hiPSCs appeared as flattened epithelial cells with distinct cell-to-cell boundaries; this was followed by a small number of spherical cells and some follicular-like cell aggregates that mostly formed at the bottom of the culture dish and floated in the culture supernatant (Fig. 1B, S2A-B). The expression of pluripotent genes *OCT4*, *SOX2*, and *PRDM14* was significantly decreased from day 0 to day 10 of differentiation (Fig. 1C, S3A). The expression of genes related to estrogen biosynthesis were determined to evaluate the synthesis of reproductive hormones during differentiation. *CYP19A1* and *CYP17A1*, and a gene for the follicle-stimulating hormone receptor (*FSHR*) were significantly increased, indicating that the granulosa-like cells within the differentiated cells produced hormones to stimulate the development of female reproductive physiology (Fig. 1C, S3A). Additionally, following 10 days of differentiation, the expression level of c-KIT increased from 0.45% in hiPSCs and 0.95% in hESCs to 50.6% and 42% in iPGCs, respectively (Fig. 1D, S3B). The interplay of c-KIT from PGCs and SCF from somatic cells can alter the adherence of PGCs to somatic cells, contributing to oogenesis [17].

**Fig. 2:**
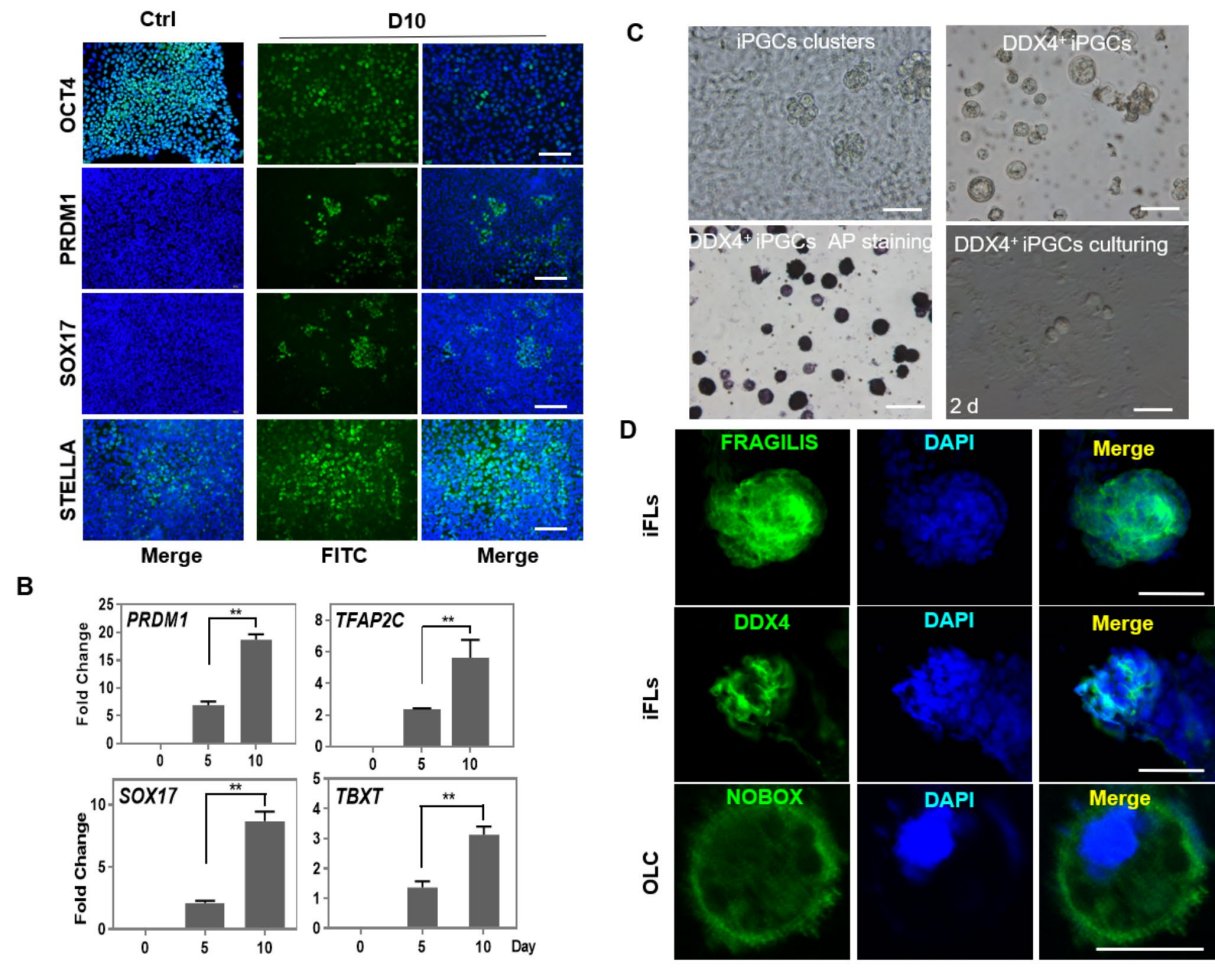
Characterization of iPGCs. (A) Immunocytochemistry images showing expression levels of OCT4, PRDM1, SOX17, and STELLA in iPGCs at day 10 (D10) of post induction. Ctrl, hiPSCs were used as control. Scale bar, 50 μm. (B) qRT-PCR analysis of PGC-related genes in iPGCs. (C) iPGCs clusters were semi-suspended above the monolayer cell at day 10 of post-induction. The iPGCs were successfully purified by the DDX4 antibody and expressed alkaline phosphatase though immunomagnetic isolation method, Mitotic cells were observed after 2 days of culture in PGCs culture system. Scale bar, 50 μm. (D) Immunocytochemistry images showing expression levels of FRAGILIS, DDX4, and NOBOX in iFLs (Scale bar, 100 μm) and OLC (Scale bar, 50 μm) at day 8 of post-induction

To further support the existence of iPGCs, immunofluorescence analysis revealed that cell clusters from both hiPSCs and hESCs expressed the crucial PGCs-related markers, including OCT4, PRDM1, SOX17, and STELLA on day 10 of differentiation (Fig. 2A, S3C). The expression of germline-specific genes *PRDM1*, *TFAP2C*, and *SOX17* was significantly higher compared with the early stage of differentiation (day 5), which was consistent with previous findings in mouse PGCs [18]. The early mesoderm marker *TBXT* (also known as *T* or *Brachyury*) was significantly activated, indicating that cells were transformed into the incipient mesoderm-like cells (iMeLCs) and activated to primordial germ cell fate [14, 19, 20] (Fig. 2B). In the late stage of iPGCs (day 10 ~ 13), some cell clusters composed of iPGCs were semi–suspended above the monolayer cells in the bottom of culture plate, and these iPGCs could be purified by DDX4 antibody after immunomagenetic isolation. The DDX4^+^ purified cells had a round-oval shape with a large nucleus with a diameter of 24.1 ± 3.82 μm, and they expressed alkaline phosphatase. Mitotic cells (with a diameter of 18.12 ± 1.66 μm) were observed after 2 days of growth in the PGCs culture system (Fig. 2C). Moreover, DDX4, BMP15, and ZP3 were expressed in DDX4 purified cells, while FRAGILIS, DDX4, and NOBOX were expressed in iFLs after 7–13 days (Fig. S2C and Fig. 2D); they are essential factors for folliculogenesis and regulation of oocyte development. These findings demonstrated that human iPSCs and ESCs could be differentiated into the late-stage PGCs and early-stage ovarian follicle via iMeLCs.

### Transcriptome Analysis of iPGCs

**Fig. 3:**
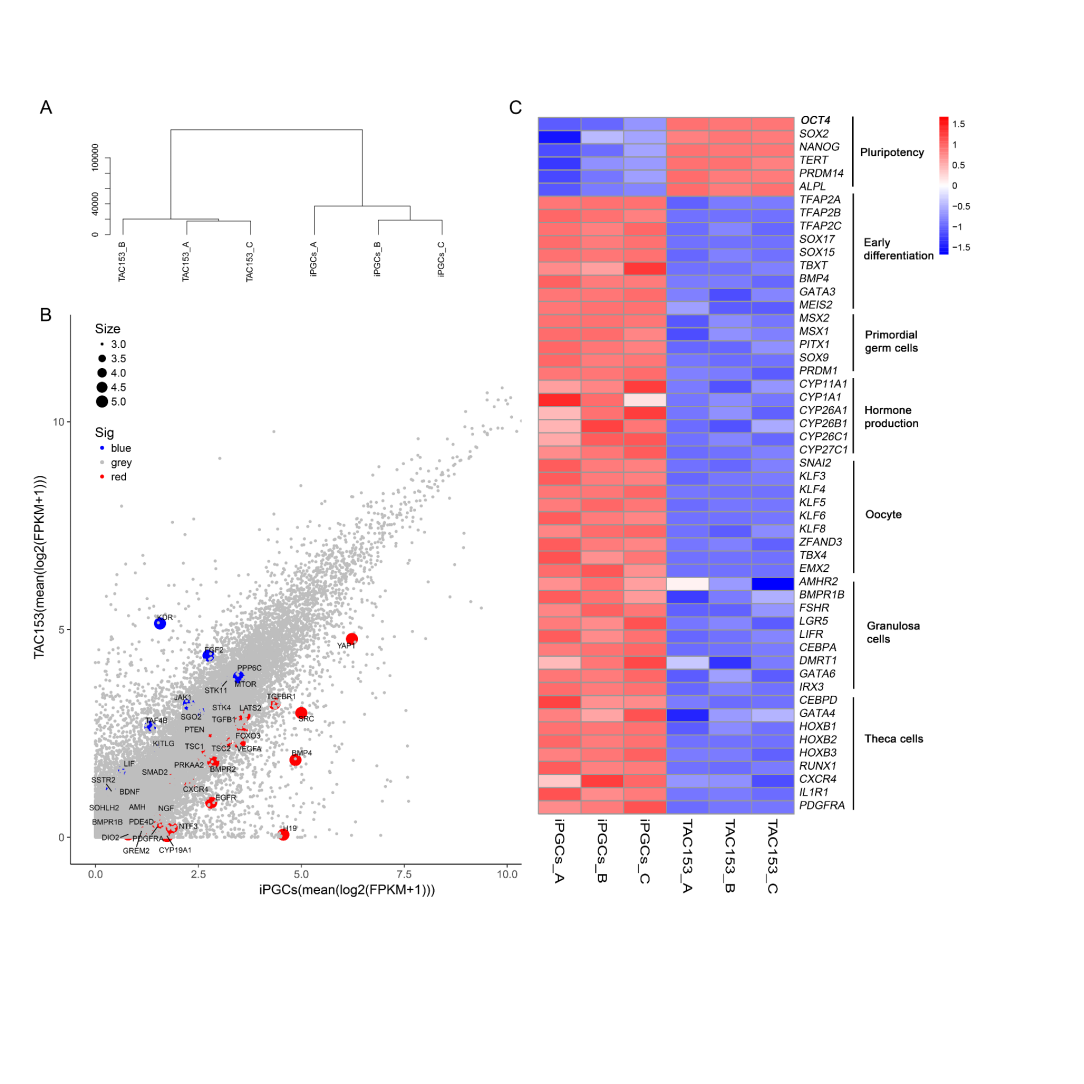
Transcriptome sequencing of iPGCs. (A) Unsupervised hierarchical clustering of gene expression in hiPSCs (TAC153) and iPGCs (D5). For each group, independent biological duplicates were subjected to whole-genome RNA sequencing. (B) Scatter plot analysis of transcripts between iPSCs and iPGCs (D5). Red dots indicating that genes enriched in reproductive cells (oocytes and granulosa cells) were highly expressed in iPGCs. Blue dots show the enriched genes in iPSCs. (C) Heat map of gene expression of iPSCs and iPGCs (D5) indicating the pluripotent stem cell and germ cell-specific differentially expressed transcripts. Red and blue indicate higher and lower levels of expression respectively. Hierarchical clustering was performed on log2 signal intensity data. These values were resized to Row Z-Score scale for any single genes (from − 1.5, the lowest expression to + 1.5, the highest expression)

To determine whether iPGCs expressed the PGC-specific markers, we performed whole-genome transcriptome sequencing to analyze of the iPSCs (TAC153) and iPGCs produced from iPSCs (D5). Unsupervised hierarchical clustering of genes showed that each group clustered to its duplicate (Fig. 3A). Principal component analysis of the transcriptome datasets combined with the Ovarian Kaleidoscope database (OKdb; http://ovary.stanford.edu), which provides information on the biological function, expression pattern, and regulation of genes expressed in the ovary, revealed that many primordial follicle growth and development associated genes, including *YAP1*, *SRC*, *BMP4*, *TGFBR1*, and *FOXO3*, etc., were extensively upregulated in iPGCs. Alternatively, mesodermal and stem cell-related genes such as *KDR*, *FGF2*, *PPP6C*, *STK11*, *LIF*, and *KITLG*, among others, were downregulated (Fig. 3B). The heat map data showed that several differentially expressed genes between the hiPSCs and iPGCs, with iPGCs consistently downregulating pluripotent genes, *OCT4, SOX2*, *NANOG*, *TERT*, PRDM14, and ALPL. Moreover, iPGCs expressed multiple early germ cells genes, including *TFAP2C*, *SOX17*, *SOX15*, and *PRDM1*, indicating the acquisition of a bona fide early germ cells fate. Although markers associated with a later germ cell fate such as *DDX4* and *DAZL*, were missing in our dataset, numerous genes involved in differentiation and reproductive processes were extensively upregulated in iPGCs, and differentiated cells displayed unique correlation with the cells in the human follicle when the whole transcriptome was compared with adult ovarian six main cell clusters [21] (Fig. 3C and Fig S2D). These data suggest after 5 days of incubation in PGC-m, the hiPSCs exited from their pluripotent state, differentiated into germ cells, and initiated folliculogenesis.

### Formation and Growth of Induced Follicle-like Structures (iFLs)

**Fig. 4:**
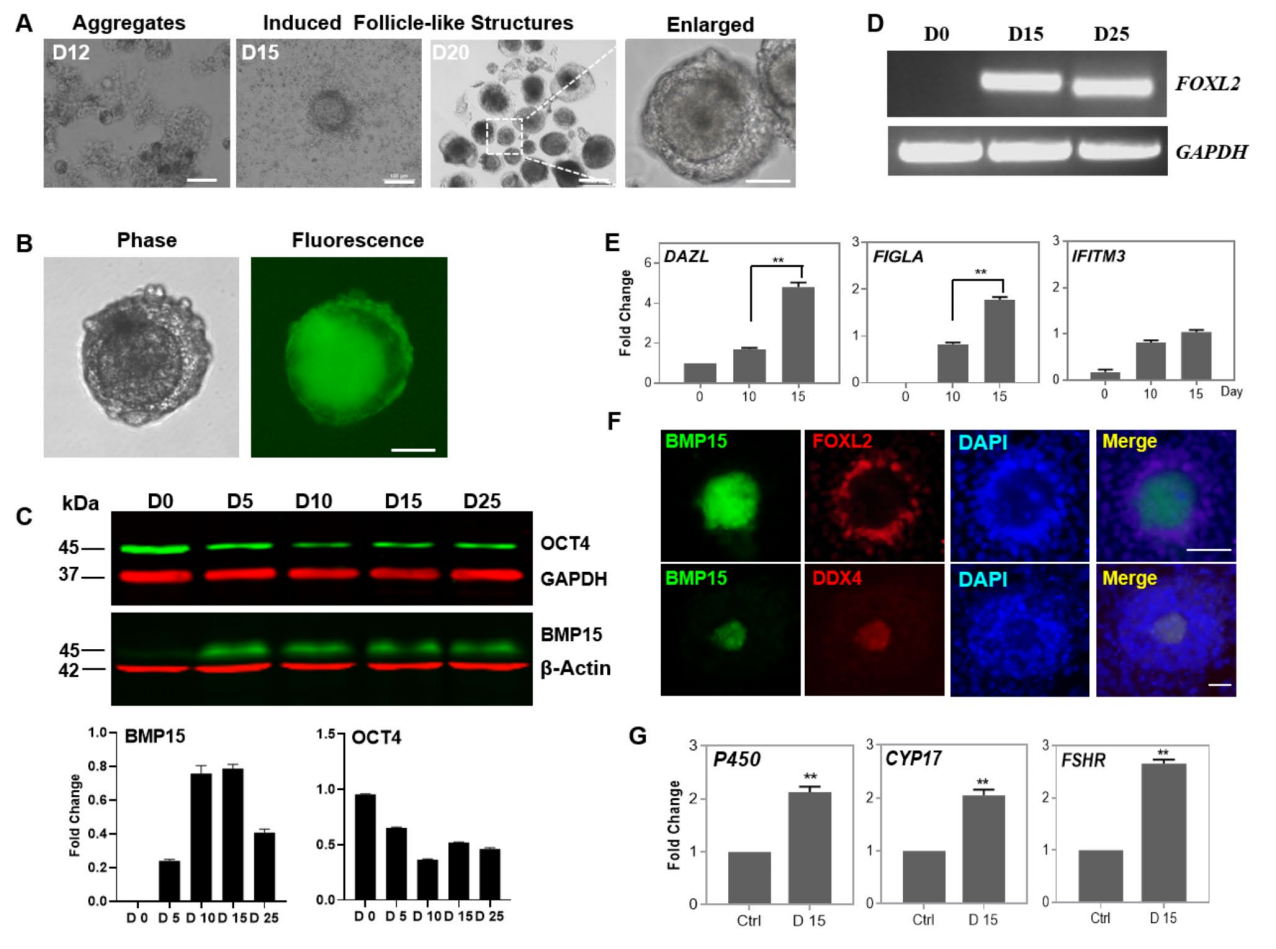
Characterization of iFLs. (A) Cell aggregates and iFLs at 12 to 20 days post-induction. (B) Expression of BMP15-EGFP fusion protein in iFLs that were transiently transfected through the pBMP15-EGFP vector. (C) Western blotting (upper) and densitometry analysis (lower) of BMP15 and OCT4 expressions in iFLs. GAPDH or β-Actin was used as internal control. (D) RT-PCR analysis of FOXL2 expression in iFLs. GAPDH served as the internal control. (E) qRT-PCR analysis of oocyte-specific markers. (F) Immunocytochemistry analysis of FOXL2 and DDX4 expressions in iFLs which were transfected with pBMP15-EGFP vector. (G) qRT-PCR analysis of hormone-related genes in iFLs at 15 days post-induction. Ctrl, human ovarian tissue as positive control. Scale bar, 50 μm for A and F; 100 μm for B. Data indicate mean ± SD. **P < 0.01, n = 3

The iFLs were formed gradually and floated in the culture medium when the induction was continued from day 10 to day 15, displaying the morphological structure of a follicle with granulosa cells and COCs (Fig. 4A, S2A-B). Several iFLs with granulosa-like cells were released from cell aggregates at this stage, and iFLs of different sizes were evident (Fig. S2A-B). Some iFLs adhered to the surface of culture plate, while others were floating in the culture medium (Fig. 4A, and video S). The pBMP15-EGFP plasmid-transfected iFL displayed green fluorescence (Fig. 4B). These results suggested that the oocyte-specific BMP15, which has high homology with GDF9 and plays crucial role in both oocyte maturation and fertilization [3; 16], was activated in the iFL. Western blot analysis validated that activation of BMP15 expression in iPGCs and iFLs stages, but OCT4 expression was considerably suppressed (Fig. 4C).

The expression of granulosa cell marker *FOXL2* in 15-day and 25-day iFL samples confirmed the presence of granulosa cells around the iFL, however, it was lacking in control samples (Fig. 4D). qRT-PCR analysis of the expression of oocyte-specific markers revealed significantly elevated levels of *DAZL*, *FIGLA*, and *IFITM3* (Fig. 4E, S3A). Furthermore, we used the BMP15-positive iFLs to perform the immunostaining with anti-FOXL2 and anti-DDX4 antibodies, as DDX4 is a unique protein in germ cell lineages [22]. These findings demonstrated that a BMP15/DDX4 positive OLC was surrounded with FOXL2 positive cells (Fig. 4F). Estrogen biosynthesis-related genes *CYP19A1*, *CYP17A1*, and *FSHR* were highly expressed in iFLs compared with the control from human ovary (Fig. 4G). These results indicated that iPGC-derived follicle-like structures expressed multiple female germline cell markers and retained the immature OLC.

### OLCs Derived from Follicles and *in vitro* Maturation

**Fig. 5:**
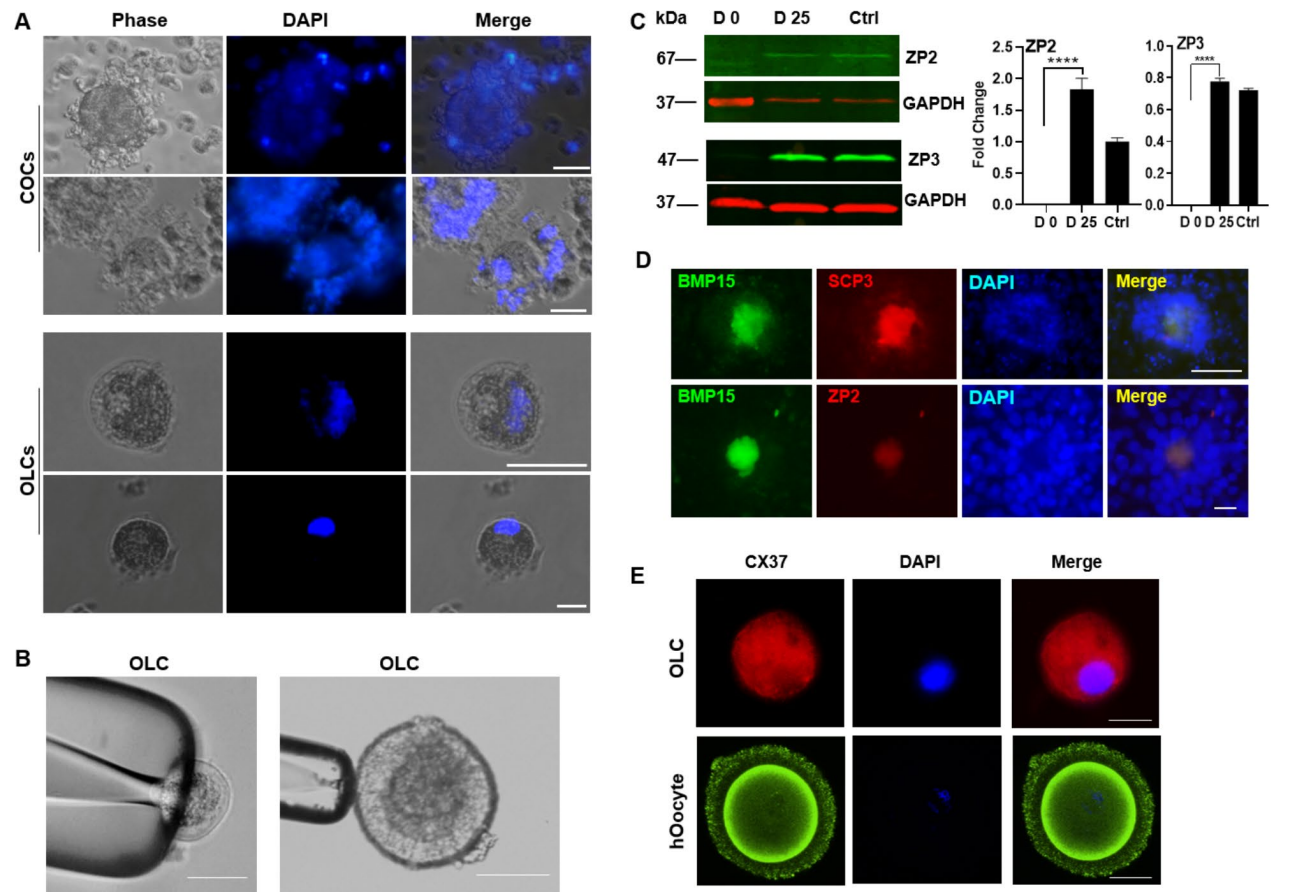
OLCs derived from iFLs. (A) COCs and OLCs derived from iFLs. The cell nuclei were stained with DAPI. (B) OLCs were held by the micromanipulator glass holding pipette. Scale bar, 50 μm (left) and 100 μm (right). (C) Western blotting (left) and densitometry analysis (right) of ZP3 expression in OLCs. GAPDH served as the internal control. (D) Immunocytochemistry analysis of SCP3 and ZP2 in OLCs which were transiently transfected with pBMP15-EGFP vector. (E) Immunocytochemistry analysis of CX37 expression in OLCs. Human oocyte (hOocyte) served as the positive control. Scale bar, 50 μm for A and D; 100 μm for E

Follicles with large nuclei were observed after 15 to 20 days of induction. The detached iFLs were harvested and cultured in OLC-m for 10 days. OLCs with diameters ranging from 50 to 150 μm coating zona pellucida (ZP) were separated from iFLs and surrounded by granulosa-like cells (Fig. 5A). Though the thin ZP was fragile and difficult to be manipulated with a micropipette, an OLC could be picked up by a holding pipette (Fig. 5B). The expression of ZP3 and BMP15 in both iPSCs and ESCs produced OLCs was confirmed by western blotting (Fig. 5C, S3E). We subsequently identified the expression of SCP3, a meiosis marker, as well as initiation of ZP glycoprotein 2 synthesis in OLCs, and verified that SCP3 and ZP2 were co-localized in OLCs but not in the surrounded granulosa-like cells (Fig. 5D). SCP3 protein and DDX4 were also found in BMP15 positive iFLs derived from hESCs (Fig. S3D). Low CX37 (Connexin 37) expression in OLCs suggested the link between granulosa cells and OLCs, as evidenced by the distribution on the whole cytoplasm when compare with human oocytes (Fig. 5E). Which mediates the formation of gap junction communication between oocyte and granulosa cells and is mainly expressed at the membrane of oocyte *in vivo* [23].

**Fig. 6:**
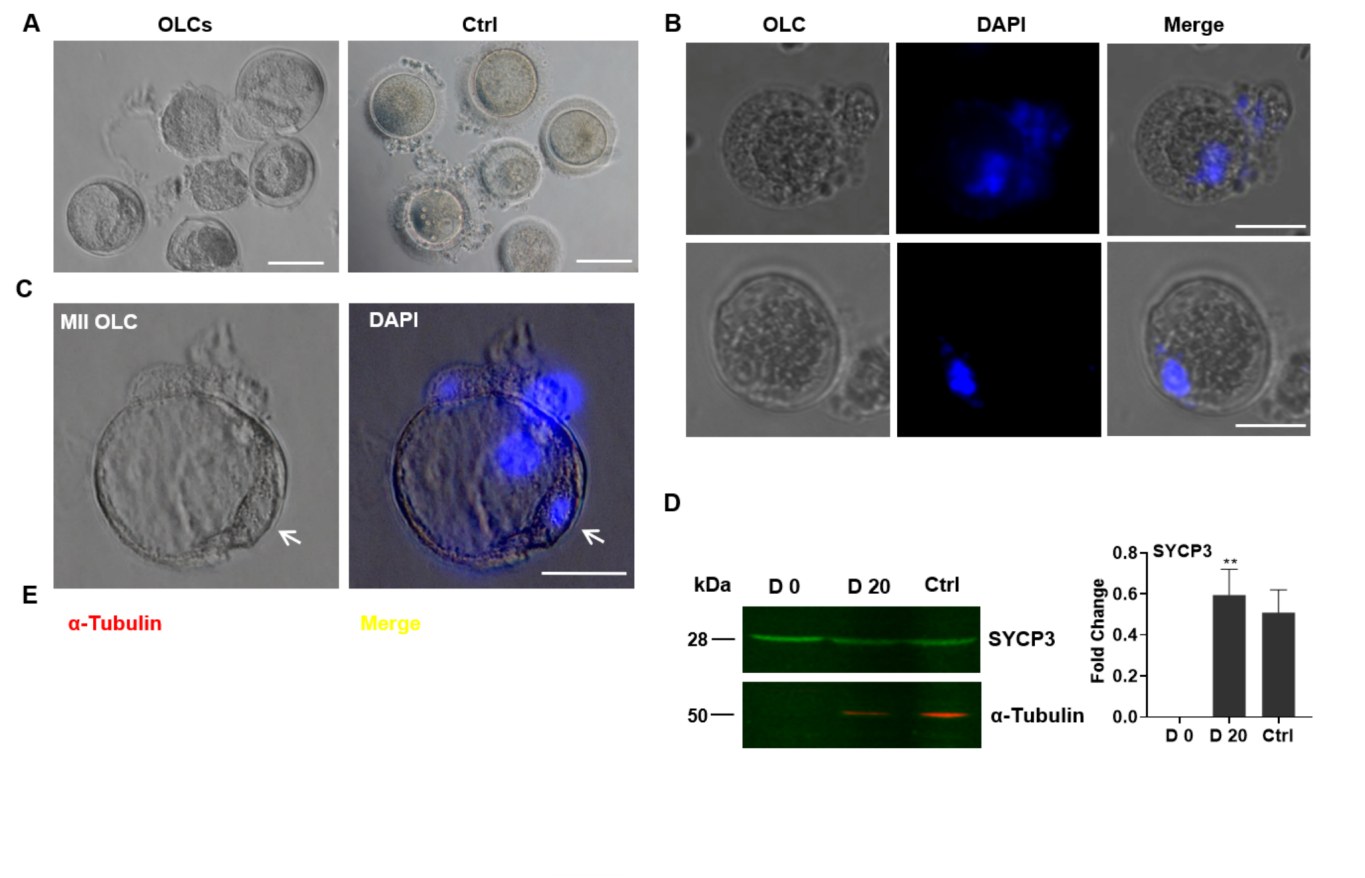
OLCs maturation *in vitro*. (A) The *in vitro* matured OLCs. Ctrl, human oocytes served as the positive control. (B) OLCs were stained with DAPI. (C) OLCs cultured in OLC-m were arrested at MII stage. Arrows indicate the first polar body. Nuclei were stained by DAPI. (D) Western blotting (left) and densitometry analysis (right) of SYCP3 expression in OLCs. Ctrl, human ovary tissue as the positive control. α-Tubulin was as internal control (E) Immunocytochemistry of α-Tubulin expression in MII OLC, the cell nuclei were stained with DAPI. Scale bar, 50 μm for B; 100 μm for A, C, and E

Germinal vesicle (GV) stage OLCs with morphologically-normal human oocytes appearance were extracted from cumulus cells 25 days after induction (Fig. 6A). DAPI staining revealed a distinct nucleus in OLC (Fig. 6B, S3F) as well as the first polar body in individual OLC (Fig. 6C). Furthermore, SYCP3, a meiosis-specific marker, was highly expression in OLCs (Fig. 6D), and MII OLCs was processed for spindle staining, the oocyte exhibited abnormal meiotic spindles with a first polar body (Fig. 6E). These data demonstrated that OLCs progressed to and were halted at metaphase II (MII) stage.

### Production of Parthenogenetic Embryo-like Structures

**Fig. 7:**
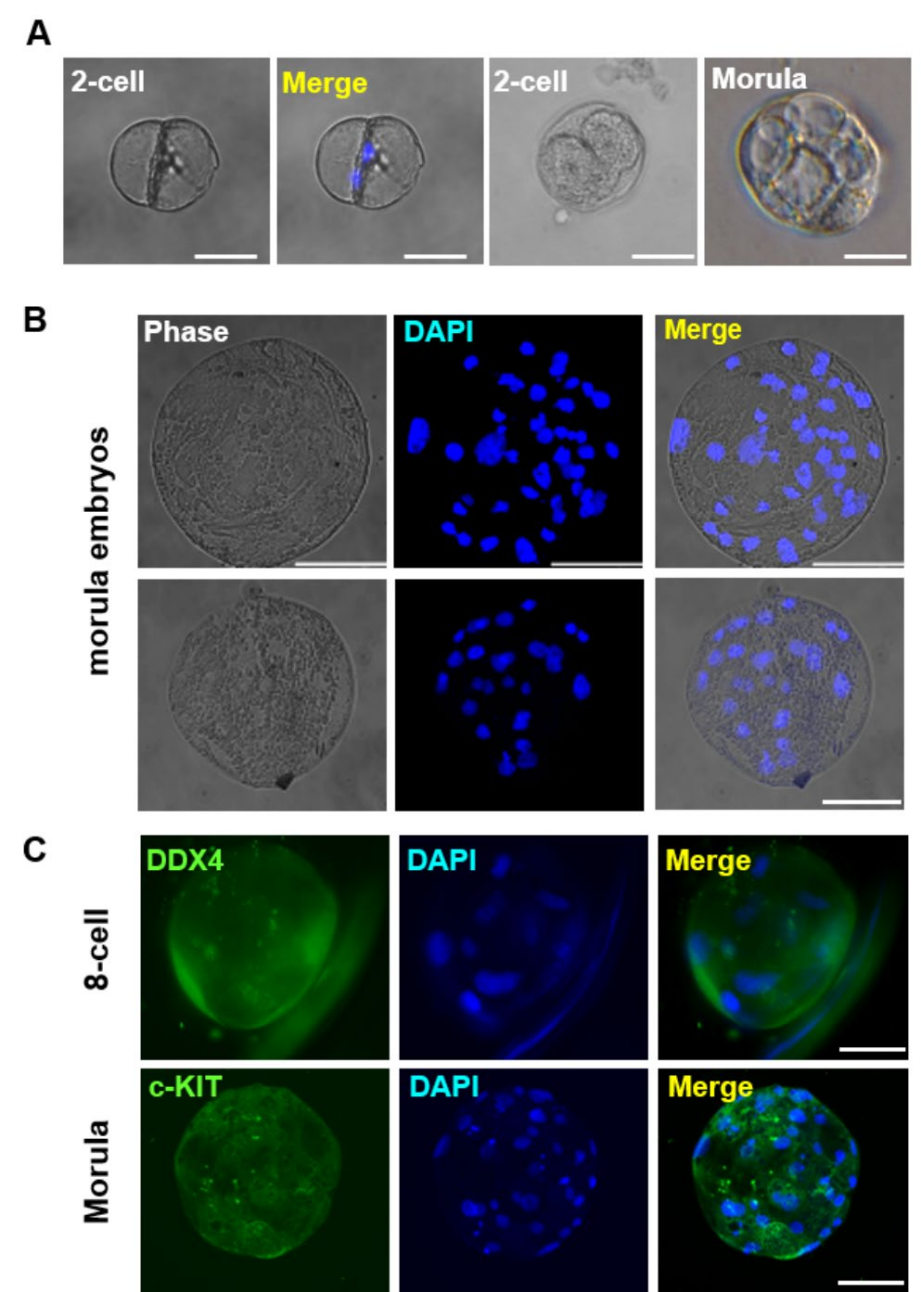
The parthenogenetic development of embryo-like structures *in vitro*. Over 30 days induction, OLCs arrested at MII stage in OLC-m showed the potential for further development due to the spontaneous parthenogenetic activation. (A) The 2-cell and early stage of morula embryos. (B) The late morula stage embryos. (C) Immunocytochemistry analysis of DDX4 and c-KIT in embryo-like structures that developed into 8-cell (DDX4) and morula (c-KIT) stages. The cell nuclei were stained with DAPI. Scale bar, 50 μm

To continue culturing OLCs up to 35 days in OLC-m, some OLCs developed spontaneously into cleavage stage-like embryos structures similar to preimplantation. The nuclear staining showed that two- and multiple nuclei were detected in embryo-like structures (Fig. 7A, S3G), indicating that OLCs were parthenogenetically activated, although these embryos was fragile with a thin ZP membrane (Fig. 7B). To elucidate the embryo-like structures, we performed IF analysis and showed that DDX4 and c-KIT were expressed in the parthenogenetically activated embryos (Fig. 7C, S3G), suggesting that DDX4 and c-KIT were involved in the progression of embryogenesis during differentiation, and parthenogenetic embryo-like structures had the development potential. Effectively regulating the parthenogenetic activation of OLCs and optimizing the culture conditions for maintaining MII type OLCs were helpful to reveal the development ability of OLCs *in vitro*.

## Discussion

This work has demonstrated a novel approach of three-step induction of hPSCs into iPGCs and OLCs in an artificial ovary without reconstitution with fetal gonadal cells. Previous research revealed that iPGCs induction was initiated after EBs formation, and ESCs or iPSCs begin spontaneous differentiation and further differentiate into iFLs and OLCs by adding cytokines and exogenous proteins [24–26]. Here, we discovered that BMP4, SCF, LIF, and EGF directly stimulated hPSCs in monolayer culture, driving iPGCs formation, and subsequently stimulated by bovine follicle fluid to form aggregates and iFLs. Under ITS and gonadotropins treatments, the percentage of postmeiotic OLCs extruded from iFLs rose and was halted at the MII stage. Although OLCs could be seen as aggregates after incubation with retinoic acid (RA) in the medium [27], the efficiency OLC generation was extremely low when follicle fluid was substituted with RA, was very low [9]. In comparison to previous methods that used mouse ESCs in monolayer culture without cytokines [28], and porcine stem cells in treatments with porcine follicular fluid, cytokines, and gonadotropin for 30–50 days [29], we found that OLCs generated in our method matured in a shorter duration and with higher efficiency without transgenes. Furthermore, early-stage OLCs with 20 ~ 50 μm expressed DDX4, BMP15, and ZP3 (Fig S3C), which were initially detected in primary oocyte in primordial follicle [30, 31]. Additionally, DDX4, a reliable marker for PGCs produced from pluripotent stem cells with high PGCs capture efficiency (42.30 ± 2.65%) [32], was found in purified cells with 16 ~ 50 μm. Yvonne and colleagues found DDX4-positive staining in both small ovarian cells (3 ~ 15 μm diameter) and oocytes in human ovarian tissue [33].

SOX17 is a critical marker in human PGC but not in mouse PGCs [14, 34]. Human iPGCs robustly induced from iPSCs via iMeLCs, which were flat epithelial cells with distinct cell-to-cell boundaries, expressed the high levels of SOX17, PRDM1, TFAP2C, and TBXT thought to be incipient mesoderm or primitive streak-like cells [14]. SOX17 and PRDM1 positive cells progressively formed clusters. These were verified in RNA-Seq data, indicating that the hPSCs differentiation approach may confer competence for germline fate [8]. However, late germ cell markers, including *DAZL*, *DDX4*, and *MAEL* were not detected in iPGCs after 5 days induction. Another intriguing observation was that the genes *GATA6* and *KITLG* revealed separate subpopulations of ovarian somatic cells [35]. As the iPGCs formed, the expression of pluripotency factors such as *OCT4*, *SOX2*, and *PRDM14* was decreased, although *PRDM14*, together with *BLIMP1* and *TFAP2C*, was considered an important factor for the specification of human PGCs [36], *PRDM14* does show a downward trend on the fifth day of induction, which was also proved by RNA-sequencing data, our data is consistent with Gell et al. [37], showing that on the fourth day of differentiation of human ESCs into PGCLCs, the down-regulation of *PRDM14* does not lose germline identity because of significantly up-regulate the PGC genes, *TFAP2C* and *PRDM1* (also known as *BLIMP1*). Along with TFAP2C and PRDM1, PGCs and gonocytes express significantly high levels of classic marker c-KIT [38]. Furthermore, whether iPGCs were induced by iPSCs or ESCs, the ovarian steroidogenic-related genes *CYP19A1*, *CYP17A1*, and *FSHR* were significantly increased, these genes have been linked to syndromic primary ovarian insufficiency (POI) [39], as have other supporting germ cell development genes such as *FIGLA*, *DAZL*, and *IFITM3*. Meanwhile, the function of other differentially-expressed genes ranged from ovarian somatic cell activation to primordial follicles formation and development, such as *YAP1* [40], *BMP4* [41] and *FOXO3* [42]. These findings imply that the formation of iPGCs is accompanied by the development of follicles.

Furthermore, we evaluated whether differentiated cells could form aggregates and generate iFLs; the iFLs retained OLCs in the middle of an aggregation surrounded by granulosa-like cells. The expressions of *DDX4*, *FRAGILIS*, *NOBOX*, *FOXL2*, *CX37*, and other genes related to female reproductive formation were validated by further investigating the existence and function of granulosa cells. Mixed cells in the aggregates were could self-organize into an ordered biological entity [43], however, in the present study, the oocyte and follicle size were relatively smaller, as well as the number of granulosa cells was lower (Fig. 5A, B and Fig S2A, B), overexpression of DAZL and BOULE in combination with cytokines GDF9 and BMP15 can induce the formation of human ovarian iFLs [3; 23]. Meanwhile, gene or protein expression shifts from OCT4 to differentiation markers (*DAZL*, *FIGLA*, *IFITM3*, BMP15, DDX4, FOXL2 and ZP3) along with the commitment of PGCs to follicular development. To evaluate OLC production, the expression of ZP2 and ZP3 was significantly elevated during OLCs formation in the later stages of induction. ZPs are membrane glycoproteins that surround the oocyte and mediate species-specific sperm binding. ZP2 functions as a secondary sperm receptor, while ZP3 is essential for sperm binding and zona matrix formation [44]. In the present investigation, *P450*, *CYP17*, and *FSHR*, which are essential for estrogen production and OLC development. Were found to be expressed during the induction. These findings demonstrate that the step induction approach produced a specific microenvironment for germ cell growth.

We discovered that bFF in PGC-m and FLC-m medium significantly increased the production of germ cells. The follicular fluid contains numerous cytokines and hormones, including GDF9 [45], bFGF [46], BMP15 [47], insulin-like growth factor [48], and gonadotropins [45]. These substances are secreted by oocytes or somatic cells and have been shown to effectively promote iFLs production and oocyte maturation [10; 16]. Although, we have studied the main components of follicular fluid in different species including human, bovine and porcine trough proteomics (data not shown), we identify a need for further research to determine which factors are likely to induce folliculogenesis. In order to understand the potential molecular mechanism of follicular fluid induced pluripotent stem cells differentiate into OLCs, single-cell epigenomic and transcriptomic analyses could provide further insights into understanding the dynamics of gene expression regulation during germ cell lineage commitment.

Recent evidence indicates that mouse ESCs may be differentiated into fully potent mature oocytes by aggregating gonadal somatic cells from mouse fetal ovaries [12], However, a comparable method is difficult to implement for hESCs, our findings demonstrated that hPSCs-derived OLCs were morphologically similar to human oocyte, this was despite the fact that OLC development was not synchronized, as evidenced by different sizes of OLCs, some of which were not surrounded by granulosa cells (below 50 μm diameter) but appeared in the first stage for iPGCs induction, whereas the larger OLCs appeared in the middle or late stages. This phenomenon might be due to granulosa cell layer proliferation and maturation or to sex hormonal regulation, which was restricted in the late stages of differentiation and OLCs development. To increase the efficiency of OLCs induction, we discovered that the master plate from which the initial run OLCs were picked up could be maintained in OLC-m and developed for an additional two weeks. During this period, run OLCs were picked up could be maintained in OLC-m and developed for an additional two weeks. During this period, OLCs were frequently released from iFLs, and OLC quality increased owing to the ZP thickness. Some OLCs with standard ZP were handled with a micropipette, while several OLCs with thin and fragile ZP were exceedingly difficult to handle with a micromanipulator. Additionally, advanced 3D culture systems can boost differentiation efficiency [49], and made iPGCs recombined with embryonic gonadal somatic cell like cells induced *in vitro* to form artificial ovary can also be improved the oocyte quality[6; 40].

## Conclusion

Whilst OLCs could reach the MII stage of post-induction, the efficiency of MII OLCs derivation was exceedingly low. The fertilization potential of OLCs in comparison to the typical eggs in vivo remains an open question. Overall, our three-step induction technique demonstrated that human MII OLCs derived from hPSCs (approximately 30 days) be employed to investigate human folliculogenesis and oogenesis *in vitro* without initial aggregation with gonad somatic cells or suspension culture to form embryoid bodies.

## Electronic Supplementary Material

Below is the link to the electronic supplementary material.

Supplementary Material 1

Supplementary Material 2

Supplementary Material 3

Supplementary Material 4

Supplementary Material 5

Supplementary Material 6

Supplementary Material 7

## Data Availability

Data supporting the current study are available from the corresponding author upon reasonable request.

## Abbreviations

OLCs: Oocyte-like cells
hiPSCs: Human induced pluripotent stem cells
hESCs: Human embryonic stem cells
PGCs: Primordial germ cells
iPGCs: Induced primordial germ cells
hPSCs: Human pluripotent stem cells
PGC-m: Primordial germ cell medium
FLC-m: Follicle like cell medium
KSR: Knockout serum replacement
bFF: Bovine follicular fluid
NEAA: Nonessential amino acids
b-ME: b-Mercaptoethanol
BSA: Bovine serum albumin
FSH: Follicle-stimulating hormone
hCG: Human chorionic gonadotropin
PMSG: Pregnant mare serum gonadotropin
EGF: Epidermal growth factor
ITS: Insulin-transferrin-selenium
cDNA: Complementary DNA
qRT-PCR: Quantitative reverse transcription PCR
GAPDH: Glyceraldehyde-3-phosphate dehydrogenase
qPCR: Quantitative polymerase chain reaction
DPBS: Dulbecco’s phosphate-buffered saline
iFLs: Induced follicle-like structures
COCs: Cumulus-oocyte-complexes
MII: Metaphase II
PGCLCs: Primordial germ cell-like cells
FLs: Follicle-like structures
FSHR: Follicle-stimulating hormone receptor
iMeLCs: Mesoderm-like cells
ZP: Zona pellucida
GV: Germinal vesicle
RA: Retinoic acid
