# Supplementary material for: Oocyte Arrested at Metaphase II Stage were Derived from Human Pluripotent Stem Cells in vitro
Source: Stem Cell Rev Rep. 2023 Feb 3;19(4):1067–81. doi: 10.1007/s12015-023-10511-7 (PMC10185642; doi:10.1007/s12015-023-10511-7)
Supplement: Supplementary file 1 — Supplementary Material 1 [file 12015_2023_10511_MOESM1_ESM.doc]

Table S1. Formulation of media used for OLC differentiation

| Media | Formulations |
| --- | --- |
| hPSCs medium | mTeSR™1 |
| PGC-medium | alpha-MEM+5%KSR+5%bFF+50ng/mL BMP4+200ng/mL LIF+100ng/mL SCF+50ng/mL EGF+10μM Y-27632+1%glutamine+1%NEAA+0.1mM b-ME |
| FLC-medium | alpha-MEM+5%KSR+5%bFF+1%L-glutamine+1%NEAA+0.1mM b-ME |
| OLC-medium | TCM-199+3mg/mL BSA+5U/mL FSH+10U/mL hCG+10IU/mL PMSG+0.23mM pyruvic acid+10ng/mL EGF+1%ITS |

Table S2 **Primers were used in this study**

| Gene | Forward primer (5’-3’) | Reverse primer (5’-3’) |
| --- | --- | --- |
| BLIMP1^a^ | CGGGGAGAATGTGGACTGGGTAGAG | CTGGAGTTACACTTGGGGGCAGC |
| CYP17^a^ | ATGGCGATCAGAAGCTGGAGAAGA | AGGAGATGACATTGGTTACCGCCA |
| DAZL^a^ | TGGCCCTTCTTTCAGTGACTTC | GACCCTAGGGGGCACTAGTAA |
| DMC1 | AGAAACATGGAATTAACGTGGCT | AAATGCAGTCAAGAATCCTGGTT |
| FIGLA^a^ | gataaaaaatctcaaccgtgg | agtcgcacctttaaggatatc |
| FOXL2 | GGTCGCACAGTCAAGGAGC | CGCGATGATGTACTGGTAGATG |
| FSHR^a^ | AGAACAAGGATCCATTCCCTGCCT | AAGCTCAGAGATTTGCCGTCTCCA |
| GAPDH | acaactttggtatcgtggaa | aaattcgttgtcataccagg |
| GAPDH^a^ | GCACCGTCAAGGCTGAGAAC | TGGTGAAGACGCCAGTGGA |
| IFITM3^a^ | CGAAACTACTGGGGAAAGGGA | ATTCATGGTGTCCAGCGAAGA |
| OCT4^a^ | GCTGGAGCAAAACCCGGAGG | TCGGCCTGTGTATATCCCAGGGTG |
| P450^a^ | ATGAATCGGGCTATGTGGACGTGT | TGGTTTGATGAGGAGAGCTTGCCA |
| PRDM14^a^ | CTACCGAGCCCGAGTGGCCTAC | TAGAGCCATCCCGGGACCGCA |
| SCP3 | tggaaaacacaacaagatca | gctatctcttgctgctgagt |
| SOX17^a^ | GAGCCAAGGGCGAGTCCCGTA | CCTTCCACGACTTGCCCAGCAT |
| SOX2^a^ | ACACCAATCCCATCCACACT | CCTCCCCAGGTTTTCTCTGT |
| T^a^ | CCTTGCTCACACCTGCAGTAGC | GGCCAACTGCATCATCTCCA |
| TFAP2C^a^ | CGCTCATGTGACTCTCCTGACATCC | TGGGCCGCCAATAGCATGTTCT |

a, Primers were used for qRT-PCR analysis.
