# Supplementary figures and images for: Oocyte Arrested at Metaphase II Stage were Derived from Human Pluripotent Stem Cells in vitro
Source: Stem Cell Rev Rep. 2023 Feb 3;19(4):1067–81. doi: 10.1007/s12015-023-10511-7 (PMC10185642; doi:10.1007/s12015-023-10511-7)

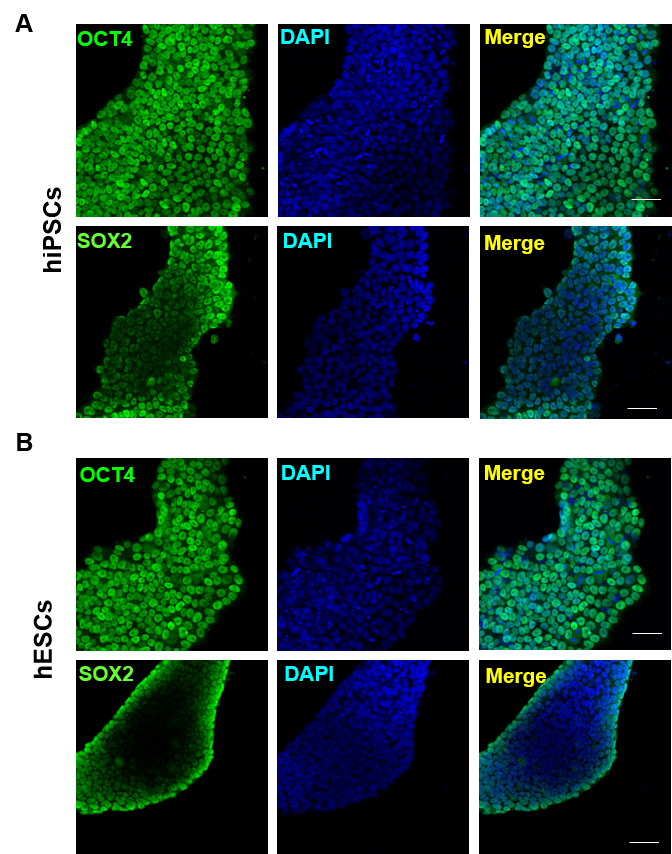

Supplement: Supplementary file 2 — Supplementary Material 2 [file 12015_2023_10511_MOESM2_ESM.tif]

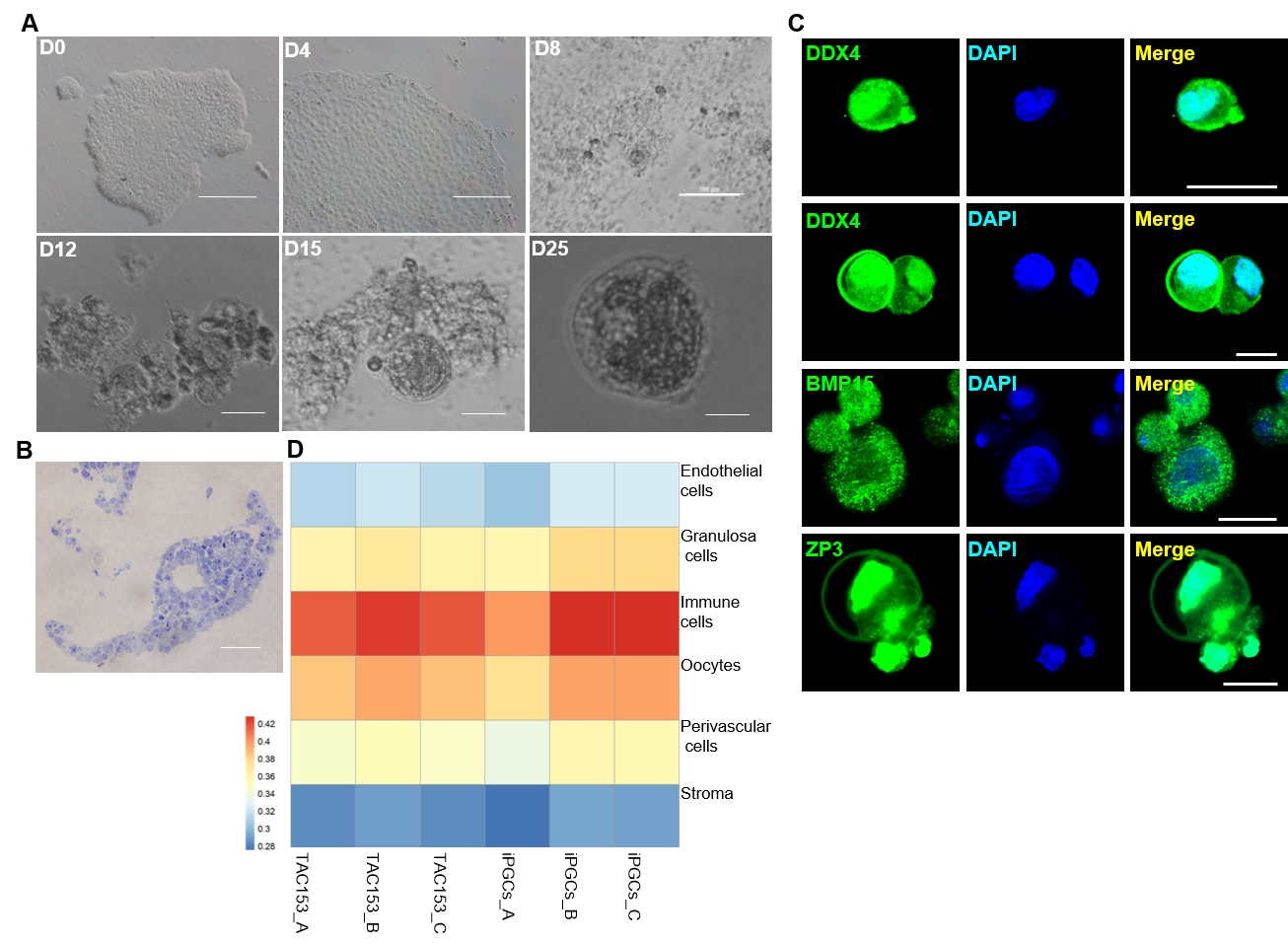

Supplement: Supplementary file 3 — Supplementary Material 3 [file 12015_2023_10511_MOESM3_ESM.tif]

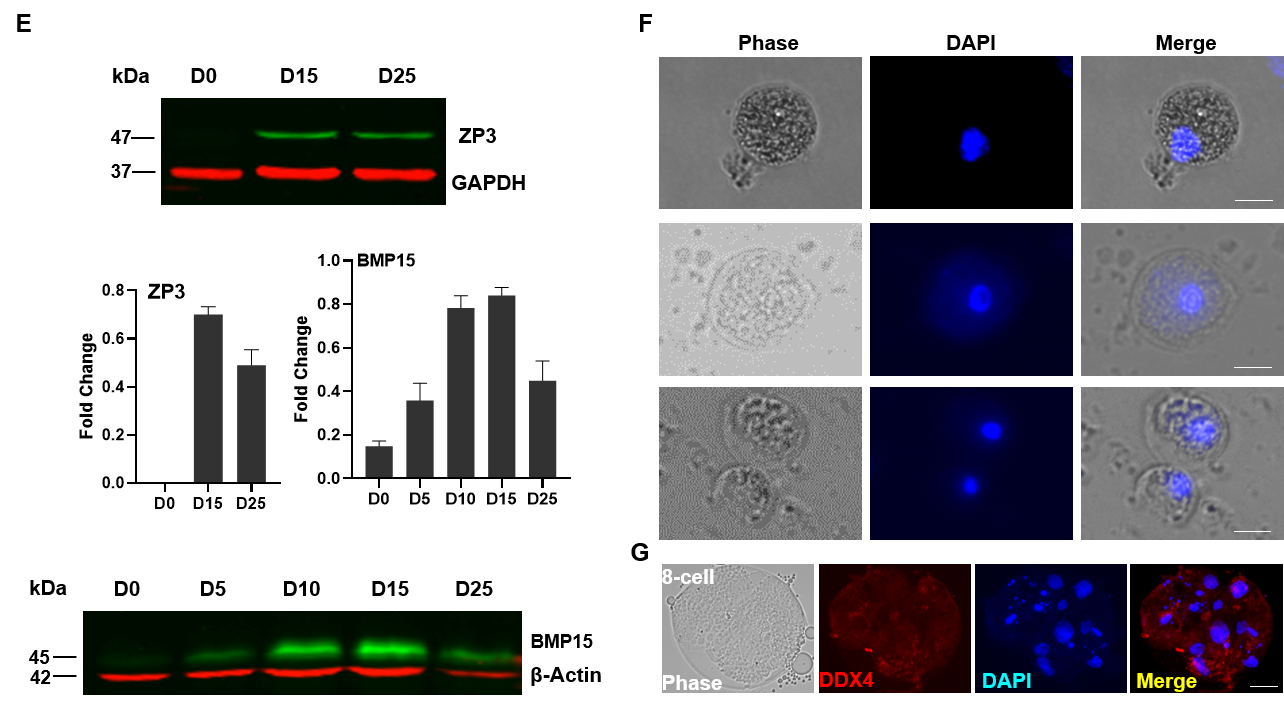

Supplement: Supplementary file 4 — Supplementary Material 4 [file 12015_2023_10511_MOESM4_ESM.tif]

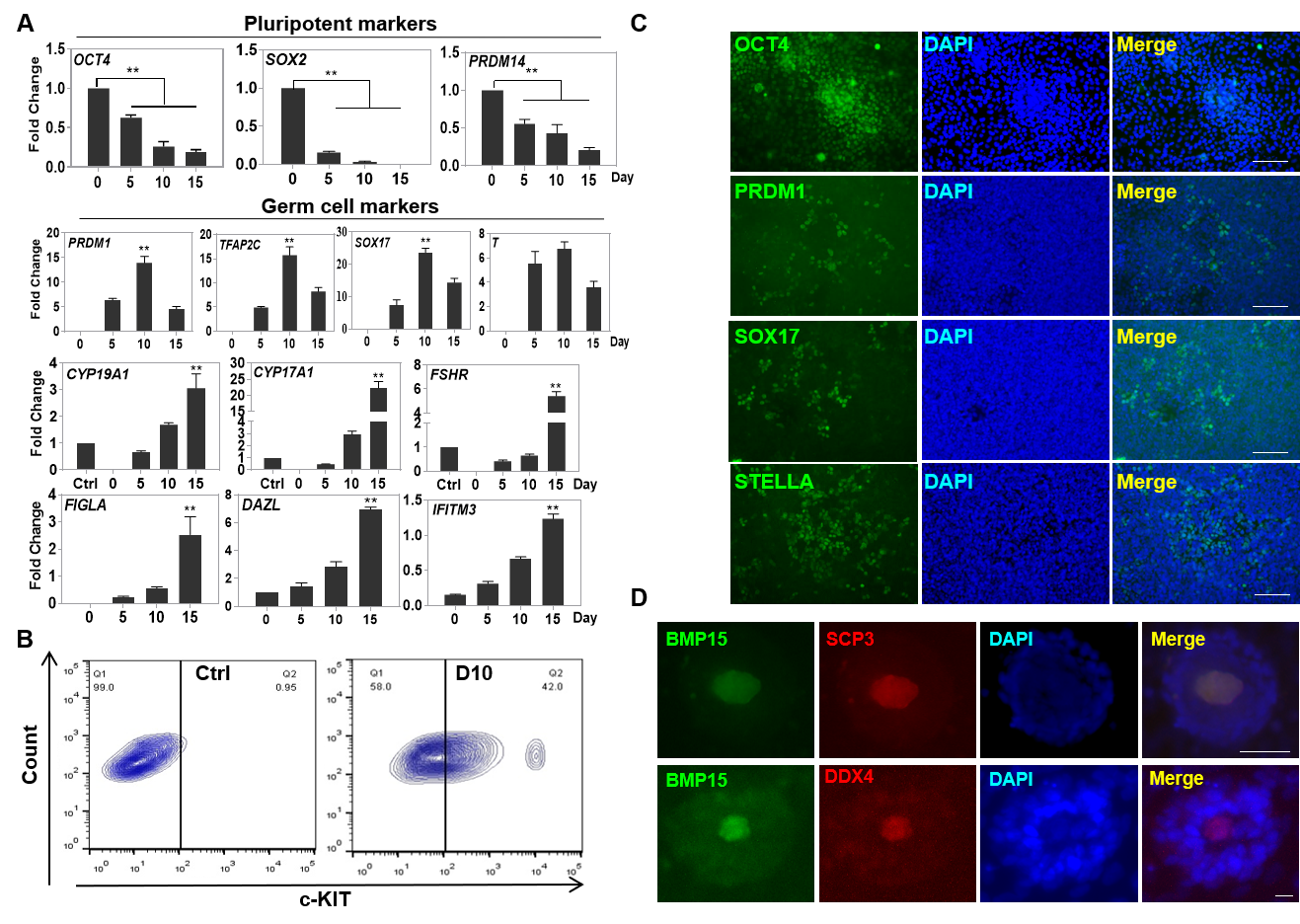

Supplement: Supplementary file 5 — Supplementary Material 5 [file 12015_2023_10511_MOESM5_ESM.tif]
