# Supplementary material for: Oocyte Arrested at Metaphase II Stage were Derived from Human Pluripotent Stem Cells in vitro
Source: Stem Cell Rev Rep. 2023 Feb 3;19(4):1067–81. doi: 10.1007/s12015-023-10511-7 (PMC10185642; doi:10.1007/s12015-023-10511-7)
Supplement: Supplementary file 6 — Supplementary Material 6 [file 12015_2023_10511_MOESM6_ESM.doc]

**Supplemental Information**

**Figure S1. Characterization of human PSCs.** **A-B** Immunocytochemistry analysis for OCT4 and SOX2 expressions in hiPSCs (A) and hESCs (B). Nuclei were stained by DAPI. Scale bar, 50 μm.

**Figure S2. The Characterization of human pluripotent stem cells during differentiation. A** Female germ cells derived from hESCs were induced for 0 day (D0) to 25 days (D25). Scale bar, 200 μm (upper) and 25 μm (down). The iFLs were observed in 10-15 days. OLCs could be further developed when cultured in OLC-m (D25). **B** The iFL from hiPSCs was differentiated for 10 days induction and were performed by semi-thin sections; bar, 50 μm. **C** Immunocytochemistry analysis for MVH, BMP15, and ZP3 expression in DDX4^+^ iPGCs after 10 days induction. Scale bar, 25 μm. **D** Heat map of whole transcriptome of iPSCs (TAC153) iPGCs (D5) compared to six main cell clusters from human adult ovarian cortex, Red and blue indicate higher and lower levels of expression respectively.

**Figure S3. Female germ cells derived from hESCs.** **A** qRT-PCR analysis of pluripotent and germ cell markers. *GAPDH* was as internal control. **B** Flow cytometry analysis of c-Kit in hESCs (Ctrl) and early stage of PGCLCs at day 10 (D10). **C** Immunocytochemistry analysis of OCT4, PRDM1, SOX17, and STELLA expressions in PGCLCs that were induced for 10 days. **D** Immunocytochemistry analysis SYCP3 and DDX4 expressions in PGCLCs that were transfected by pBMP15-EGFP vector. **E** Western blotting (left) and densitometry analysis (right) of ZP3 and BMP15 expressions. GAPDH was used as internal control. **F** OLCs were stained by DAPI. **G** Immunocytochemistry analysis of the early stage of morula embryo, which was activated due to the spontaneous parthenogenesis, was detected by anti-DDX4 antibody. Nuclei were stained by DAPI. Scale bar, 50 μm. Data indicate mean ± SD. ***P*<0.01, n=3.
